# Cleomin Exerts Acute Antinociceptive Effects in Mice via GABA_B_ and Muscarinic Receptors

**DOI:** 10.3390/ph16111547

**Published:** 2023-11-02

**Authors:** Luíza Carolina França Opretzka, Max Denisson Maurício Viana, Alyne Almeida de Lima, Thalisson Amorim de Souza, Marcus Tullius Scotti, Josean Fechine Tavares, Marcelo Sobral da Silva, Milena Botelho Pereira Soares, Cristiane Flora Villarreal

**Affiliations:** 1 School of Pharmacy, Federal University of Bahia, Salvador 40170115, BA, Brazil; luizacfo@ufba.br (L.C.F.O.); max.viana@ufba.br (M.D.M.V.); 2 Gonçalo Moniz Institute, FIOCRUZ, Salvador 40296710, BA, Brazil; alyne.lima@fiocruz.br (A.A.d.L.); milena.soares@fiocruz.br (M.B.P.S.); 3 Institute for Research on Drugs and Medicines, Federal University of Paraíba, João Pessoa 58059900, PB, Brazil; thalisson.amorim@ltf.ufpb.br (T.A.d.S.); mtscotti@ccae.ufpb.br (M.T.S.); josean@ltf.ufpb.br (J.F.T.); marcelosobral@ltf.ufpb.br (M.S.d.S.); 4 Institute of Advanced Systems in Health, SENAI CIMATEC, Salvador 41650010, BA, Brazil

**Keywords:** cleomin, antinociception, GABAergic receptor, cholinergic receptor, pain, *Neocalyptrocalix longifolium*

## Abstract

Cleomin, a 1,3-oxazolidine-2-thione, was recently isolated from *Neocalyptrocalyx longifolium*, a species traditionally used for treating painful conditions. Reports about the pharmacological activities of cleomin are lacking. Here, the antinociceptive effects of cleomin were investigated using mice models of pain, namely the formalin, the cold plate, and the tail flick tests. Motor integrity was assessed in the rota-rod test. Antagonism assays and in silico docking analyses were performed to investigate the putative mechanisms of action. Cleomin (12.5–25 mg/kg), at doses that did not induce motor impairment, induced dose-dependent antinociception in both early and late phases of the formalin test and reduced nociceptive behaviors in both the cold plate and tail flick tests. Pretreatments with phaclofen and atropine attenuated the antinociceptive effects of cleomin, implicating the involvement of GABA_B_ and muscarinic receptors. In silico docking studies suggested satisfactory coupling between cleomin and GABA_B_ and M_2_ receptors, hence corroborating their role in cleomin’s activity. Pretreatments with naloxone, yohimbine, bicuculline, and methysergide did not affect the antinociception of cleomin. In silico pharmacokinetics prediction showed a good drug ability profile of cleomin. In conclusion, cleomin promoted antinociception mediated by GABA_B_ and muscarinic receptors. These findings support further investigation of the analgesic potential of cleomin.

## 1. Introduction

The proper pharmacological control of pain is an unsolved challenge for patients and healthcare providers. Current pharmacological strategies rely on opioid analgesics and non-steroidal anti-inflammatory drugs, which present restrictions related to their safety profile and risk of abuse [1,2]. The growing global prevalence of chronic pain and the escalating opioid epidemic have highlighted the need for a paradigm shift in pain pharmacotherapy, which will require the development of new safe and effective analgesic drugs acting through different mechanisms of action [3].

Preparations derived from medicinal plants have been used for pain control for millennia. In recent years, researchers have identified several bioactive compounds that confer medicinal properties on plants. Among these compounds, 1,3-oxazolidine-2-thiones comprise a group of secondary metabolites that protect plants against stressors [4]. 1,3-Oxazolidine-2-thiones promote both anti-inflammatory [5] and antiviral [6] effects in vitro. These studies revealed that the stereochemistry of the chiral carbon significantly influences the biological activity of 1,3-oxazolidine-2-thiones. This chemical feature allows for interactions with multiple active sites, such as enzymes and membrane receptors, thus suggesting a potentially wide range of biological activities [7]. Although 1,3-oxazolidine-2-thiones promote different biological effects, their pharmacological potential is still poorly explored.

**Figure 1:**
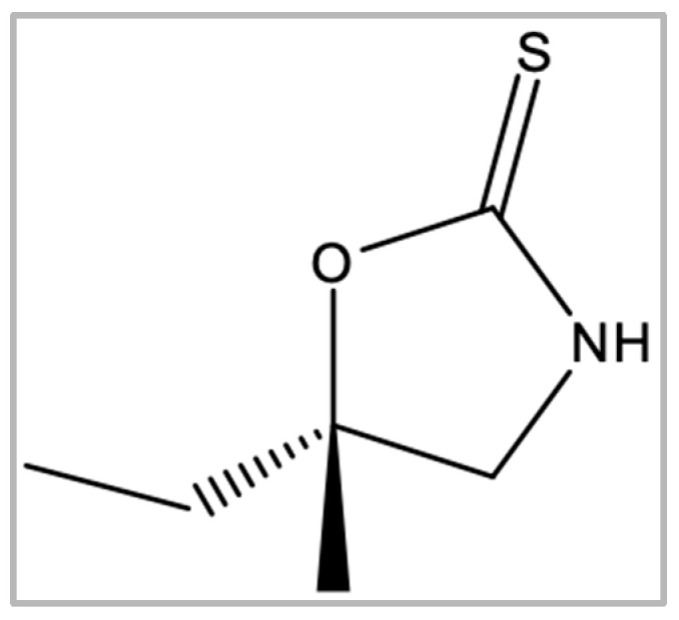
Chemical structure of cleomin.

Cleomin (5-ethyl-5methyl-oxazolidine-2-thione; Figure 1) is a 1,3-oxazolidine-2-thione that was first described in 1981 [8]. This metabolite occurs in some species of the Capparaceae family (e.g., *Neocalyptrocalix longifolium*). Recently, our group isolated cleomin from the roots of *N. longifolium* (“incó”), a plant found in the Brazilian Caatinga biome [9]. Ethnopharmacological studies report that *N. longifolium* is used for treating health ailments, including painful conditions [10]. Although at least part of these effects can potentially be attributed to cleomin, the pharmacological properties of this compound have not been experimentally investigated. The present work was designed to test the hypothesis that cleomin exerts antinociceptive action. We also investigated possible mechanisms of action for such activity using classic in vivo pain models and in silico analysis.

## 2. Results

### 2.1. Effect of Cleomin on Motor Function in Mice

**Figure 2:**
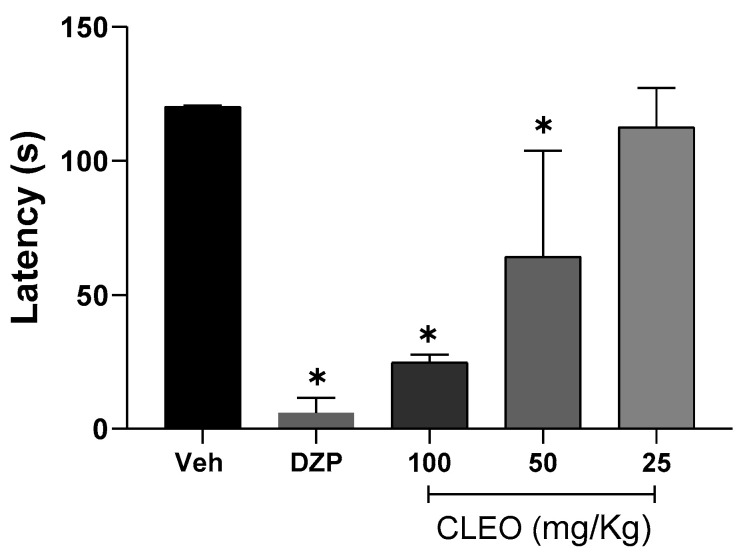
Effects of cleomin on motor function in mice. Bar graphs representing the run time on the rota-rod (in seconds) 40 min after intraperitoneal treatments with cleomin (CLEO; 25–100 mg/kg), vehicle (Veh; 5% DMSO in 0.9% saline; control group)**,** or diazepam as a reference drug (DZP; 10 mg/kg). Data are reported as means ± SD; *n* = 6 mice per group. * Different from control group (*p* < 0.05), as determined via one-way ANOVA followed by Tukey’s test.

Considering that *N. longifolium* promotes sedative effects [10], before starting the behavioral tests of nociception, the effects of cleomin on the motor function of mice were evaluated in the rota-rod test. For this test, cleomin was used intraperitoneally at doses of 100, 50, and 25 mg/kg, as shown in Figure 2. Motor function was impaired at a dose of 50 mg/kg (*p* < 0.05). As expected, the central nervous system depressant diazepam (10 mg/kg, ip), used as a standard drug, reduced the permanence time of mice on the rota-rod (*p* < 0.001). Based on these results, this study was then conducted with doses no greater than 25 mg/kg.

### 2.2. Effect of Cleomin on Pain-like Behavior in the Formalin Test

**Figure 3:**
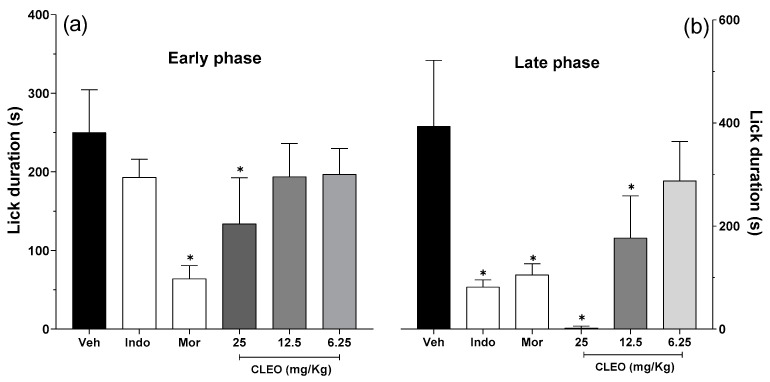
Effects of cleomin on the nociception induced by formalin in mice. On the formalin test, the pain-like behaviors exhibited by mice were quantified in the early phase (0–10 min, panel (**a**)) and late phase (10–30 min, panel (**b**)) of the test. Mice were intraperitoneally treated with cleomin (CLEO; 6.25–25 mg/kg) or vehicle (Veh; 5% DMSO in 0.9% saline; control group) 40 min before the intraplantar injection of formalin (2.5%, 20 μL). Indomethacin (Indo; 10 mg/kg) and morphine (Mor; 5 mg/kg) via intraperitoneal route 40 min before formalin were the reference drugs. Data are reported as means ± SD; n = 6 mice per group. * Different from the control group (*p* < 0.001), as determined via one-way ANOVA followed by Tukey’s test.

The antinociceptive properties of cleomin were initially investigated in the formalin test in mice. The administration of formalin in control mice induced a biphasic paw-licking response, with the early phase ranging from 0 to 10 min and late phase from 10 to 30 min after the injection (Figure 3a and Figure 3b, respectively). In the early phase of the test, the pretreatment with cleomin at 25 mg/kg diminished the nociception caused by formalin (*p* < 0.001), indicating potential analgesic activity (Figure 3a). The late phase of the test was inhibited by cleomin at doses of 12.5 and 25 mg/kg (*p* < 0.001) (Figure 3b). Cleomin at 6.125 mg/kg did not induce antinociception in the formalin test. Pretreatment with indomethacin (10 mg/kg, ip), a standard nonsteroidal anti-inflammatory drug, reduced the late phase (*p* < 0.001), while the pretreatment with morphine (5 mg/kg, ip), a gold-standard opioid, inhibited both the early and late phases (*p* < 0.001) of the formalin test. Therefore, the inhibition profile of both phases suggests that cleomin mediates its effects through the modulation of pain transmission rather than through anti-inflammatory action.

### 2.3. Cleomin Reduces Pain-like Behavior in Cold Plate and Tail Flick Tests

**Figure 4:**
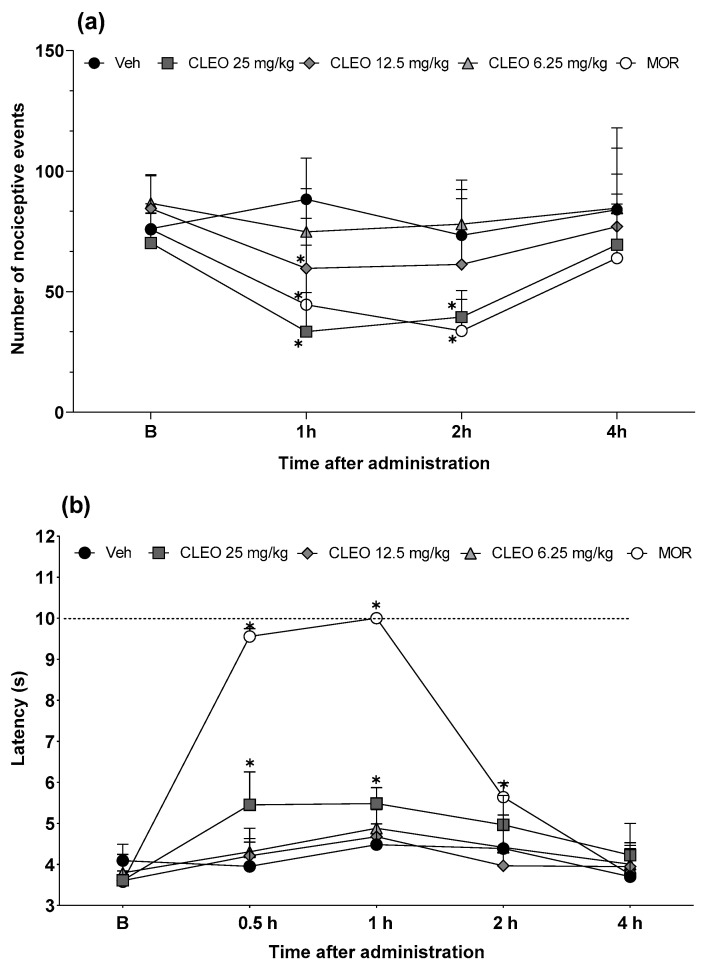
Effects of cleomin treatment on cold plate and tail flick tests in mice. Panels representing the number of nociceptive events on cold plate test (**a**) and the latency in seconds in the tail flick test (**b**) after intraperitoneal administration of cleomin (CLEO; 6.25–25 mg/kg), vehicle (Veh; 5% DMSO in 0.9% saline; control group), or morphine (MOR; 5 mg/kg; reference drug) at different times. Baseline measures (B) are represented in both panels. The dashed line represents the cutoff time (10 s). Data are reported as means ± SD; n = 6 mice per group. * Different from control group (*p* < 0.05), as determined via two-way ANOVA followed by Bonferroni’s post-test.

To investigate the possible central mediation in cleomin-induced antinociception, the effects of this compound were also evaluated in the cold plate and tail flick tests, which mainly identify central analgesics. Figure 4a,b show the effects of cleomin in the cold plate and tail flick tests, respectively. In the cold plate test, the intraperitoneal administration of cleomin significantly reduced the number of nociceptive events, compared to the control group, at 1 h (12.5 and 25 mg/kg, *p* < 0.05) and 2 h (25 mg/kg, *p* < 0.05) after treatment (Figure 4a). In the tail flick test, the inhibition of the nociceptive response to the thermal stimulus was observed in mice treated with the highest dose of cleomin (25 mg/kg, i.p.; *p* < 0.05), at 0.5 and 1 h after treatment (Figure 4b). Cleomin at 6.25 mg/kg (i.p.) did not alter the nociceptive response in either test. As expected, the reference drug morphine (5 mg/kg, i.p.) inhibited the nociceptive responses in both tests (*p* < 0.05). These results are consistent with those observed in the formalin test.

### 2.4. Cleomin Induces Antinociceptive Effects in Mice via GABA_B_- and Muscarinic Receptors

**Figure 5:**
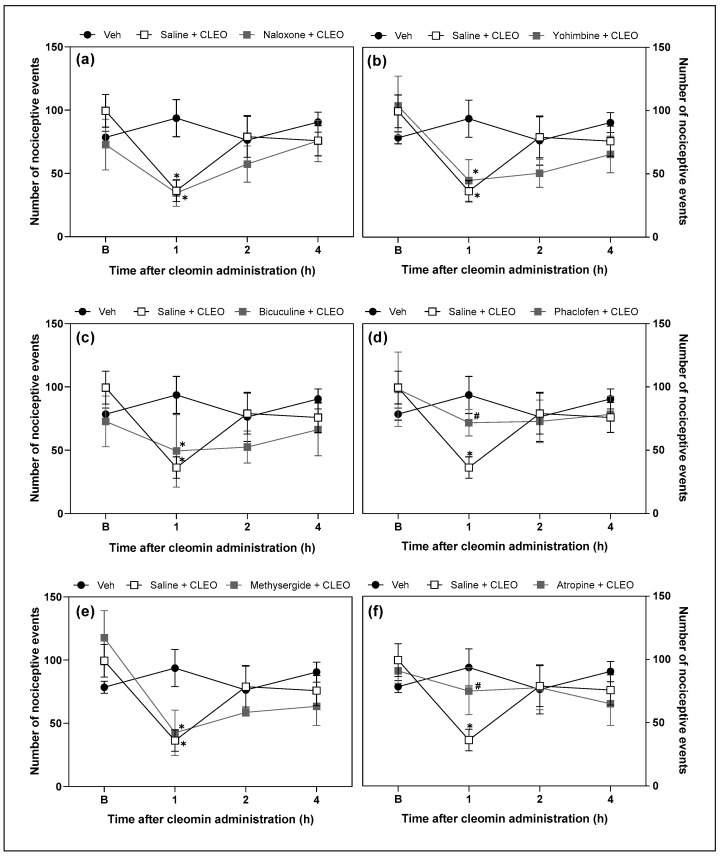
Effects of the pharmacological blockade of different receptors on the cleomin-induced antinociception on cold plate test. Panels representing the number of nociceptive events before (B), 1, 2, and 4 h after the intraperitoneal administration of cleomin (CLEO). All antagonists were administered intraperitoneally before cleomin treatment (25 mg/kg). Panel (**a**): mice were treated with naloxone (5 mg/kg), a non-selective antagonist of opioid receptors, 15 min before cleomin. Panel (**b**): mice were treated with yohimbine (2 mg/kg), a selective α2-adrenergic antagonist, 30 min before cleomin. Panel (**c**): mice were treated with bicuculline (1 mg/kg), a gamma aminobutyric acid-A GABA_A_ receptor antagonist, 15 min before cleomin. Panel (**d**): mice were treated with phaclofen (2 mg/kg), a gamma aminobutyric acid-B GABA_B_ receptor antagonist, 15 min before cleomin. Panel (**e**): mice were treated with methysergide (5 mg/kg), a non-selective serotonergic antagonist, 30 min before cleomin. Panel (**f**): mice were treated with atropine (10 mg/kg), a non-selective cholinergic antagonist, 15 min before cleomin. The Saline + CLEO group represents mice that received an intraperitoneal administration of saline before cleomin (25 mg/kg), while the Veh group mice treated with vehicle (5% DMSO in 0.9% saline) instead of cleomin. Data are reported as means ± SD; n = 6 mice per group. * Different from the Veh group (*p* < 0.05); ^#^ Different from the Saline + CLEO group (*p* < 0.05), as determined via one-way ANOVA followed by Tukey’s test.

**Figure 6:**
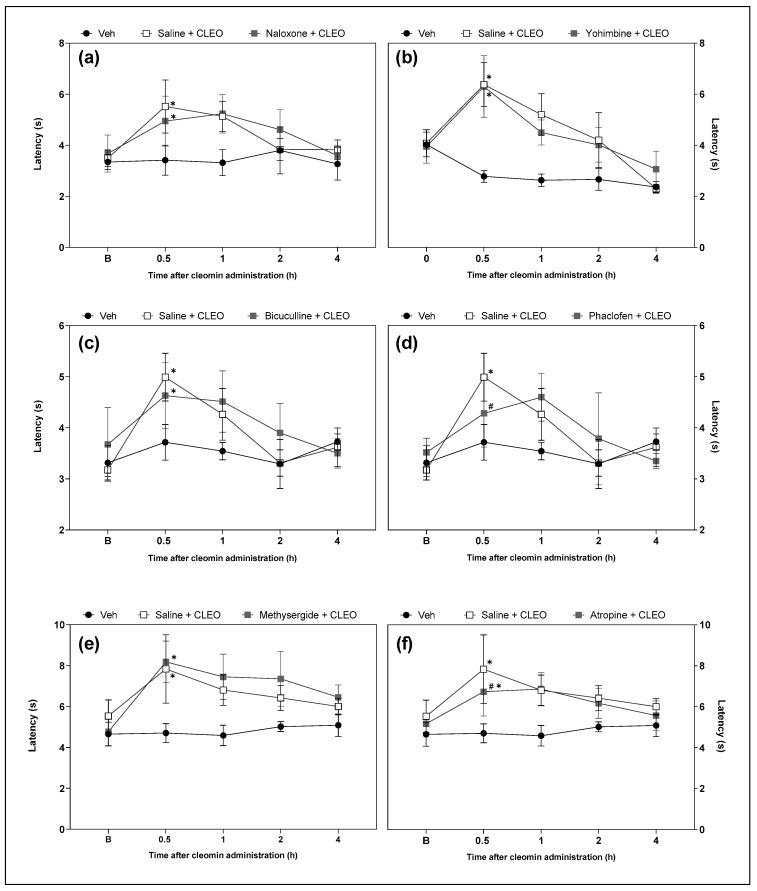
Effects of the pharmacological blockade of different receptors on the cleomin-induced antinociception on tail flick test. Panels representing the tail flick response in seconds before (B), 0.5, 1, 2 and 4 h after the intraperitoneal administration of cleomin (CLEO). All antagonists were administered intraperitoneally, before cleomin (25 mg/kg), according to the following protocol. Panel (**a**): mice were treated with naloxone (5 mg/kg), a non-selective antagonist of opioid receptors, 15 min before cleomin. Panel (**b**): mice were treated with yohimbine (2 mg/kg), a selective α2-adrenergic antagonist, 30 min before cleomin. Panel (**c**): mice were treated with bicuculline (1 mg/kg), a gamma aminobutyric acid-A GABA_A_ receptor antagonist, 15 min before cleomin. Panel (**d**): mice were treated with phaclofen (2 mg/kg), a gamma aminobutyric acid-B GABA_B_ receptor antagonist, 15 min before cleomin. Panel (**e**): mice were treated with methysergide (5 mg/kg), a non-selective serotonergic antagonist, 30 min before cleomin. Panel (**f**): mice were treated with atropine (10 mg/kg), a non-selective cholinergic antagonist, 15 min before cleomin. The Saline + CLEO group represents mice that received an intraperitoneal administration of saline before cleomin (25 mg/kg), while the Veh group mice treated with vehicle (5% DMSO in 0.9% saline) instead of cleomin. Data are reported as means ± SD; n = 6 mice per group. * Different from Veh group (*p* < 0.05); ^#^ Different from the Saline + CLEO group (*p* < 0.05) as determined via one-way ANOVA followed by Tukey’s test.

Functional antagonism assays were performed to investigate the possible role of opioid, α2-adrenergic, GABAergic (A and B), serotoninergic, and cholinergic receptors in cleomin-induced antinociception. Intraperitoneal pretreatment with phaclofen (a selective GABA_B_ receptor antagonist; 2 mg/kg) and atropine (a cholinergic receptors antagonist; 10 mg/kg), but not with yohimbine (an α2-adrenergic receptor antagonist; 2 mg/kg), bicuculline (a selective GABA_A_ receptor antagonist; 1 mg/kg), methysergide (a serotonergic receptors antagonist; 5 mg/kg), or naloxone (an opioid receptors antagonist; 5 mg/kg), attenuated cleomin-induced antinociception in the cold plate and tail flick models (Figure 5 and Figure 6, respectively).).

### 2.5. Cleomin Presented Satisfactory Coupling with GABA_B_ and M2 Receptors in Docking Analysis

**Figure 7:**
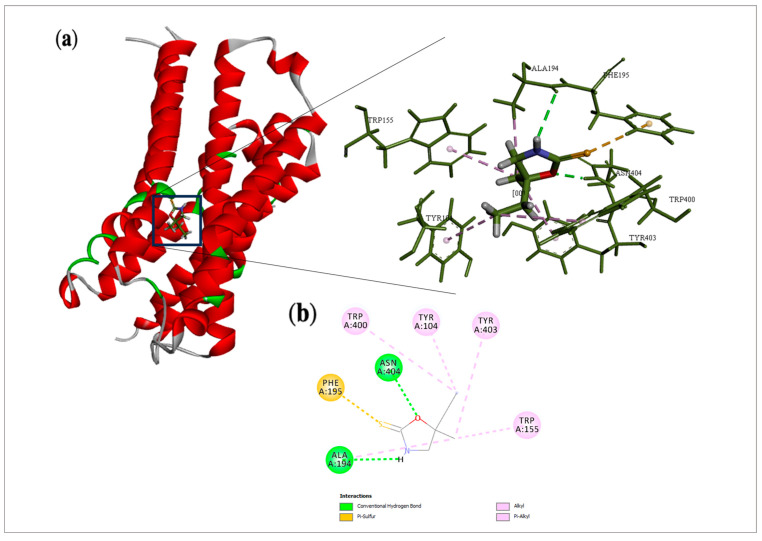
Docking analysis of cleomin in M2 receptor: (**a**) Three-dimensional structure of selected protein (PDB: 4MQS) and their interactions with cleomin. (**b**) 2D interactions of cleomin with amino acid residues in the M2 receptor.

Docking is a useful tool to elucidate the molecular aspects involved in the biological activity of compounds [11]. In the present study, it allowed us to measure the probability of interaction between cleomin and the M2 and GABA_B_ receptors. Intermolecular forces acting over cleomin and the target may enable activation of the studied receptors. The analysis in the muscarinic receptor showed that cleomin obtained an E_N_ score = −0.54, whereas iperoxo, an ortostheric super-agonist used as a crystallographic ligand of this receptor, achieved an E_N_ score = −0.69. Both compounds shared similar interactions with critical amino acid residues of M2. Among them, the presence of the oxygen atom in the oxazolidine scaffold allowed for the formation of hydrogen bonding with Asn 404 and Ala 194. In addition, Pi-alkyl interactions were also observed with Trp 155, Trp 400, Tyr 104, and Tyr 403. Differing from the crystallographic ligand, a Pi-sulfur interaction with Phe195 was found (Figure 7).

**Figure 8:**
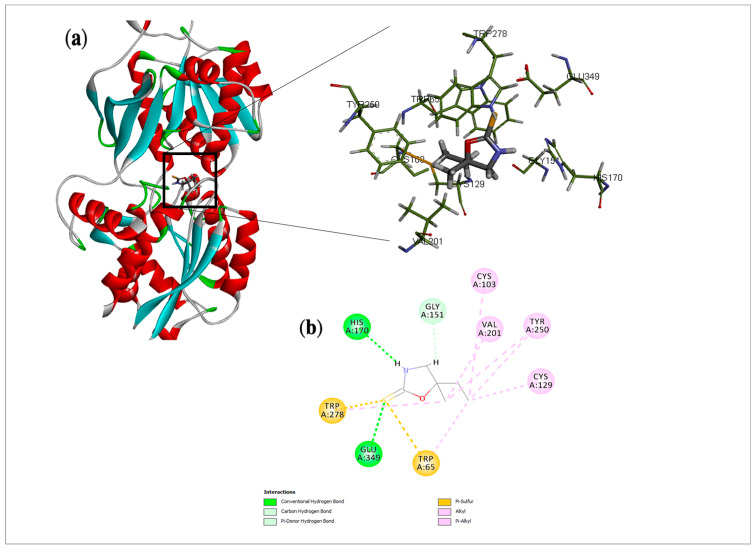
Docking analysis of cleomin in GABA_B_ receptor: (**a**) Three-dimensional structure of selected protein (PDB: 4MS3) and their interactions with cleomin. (**b**) 2D interactions of cleomin with amino acid residues in the GABA_B_ receptor. Based on the profile of interactions, the molecular docking results suggest a possible activity of cleomin over both macromolecular targets, especially in GABA_B_.

Regarding the GABA_B_ receptor, the binding mode of cleomin was compared with γ-amino-butanoic acid, the endogenous ligand for this receptor. Docking analysis showed common hydrogen bonding with Hys 170 and Glu 349. In addition, Pi and alkyl interactions were observed for Trp 65 and Trp 278 with the thione group, Val 201 and Cys 129 with alkyl and ethyl moieties, suggesting that these amino acids could mediate critical interactions with cleomin, enabling the anchoring of the compound at the active site of this receptor (Figure 8). However, the crystallographic inhibitor amino-butanoic acid displayed additional hydrogen bonding mediated by the terminal carboxylic acid moiety. The amino-butanoic acid had an E_N_ score = −0.38, whereas cleomin achieved an E_N_ score = −0.33.

### 2.6. In Silico Prediction of Cleomin Pharmacokinetics

**Figure 9:**
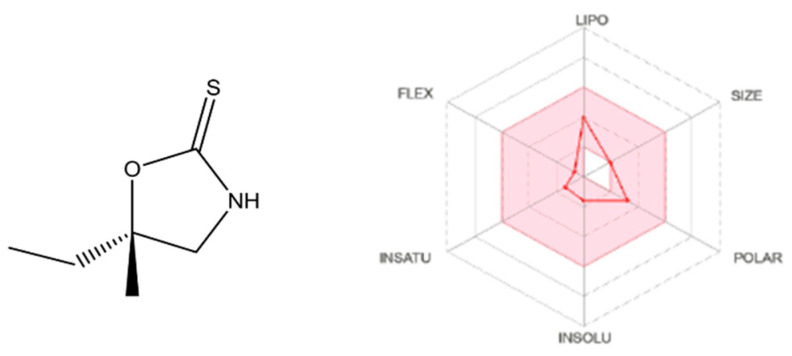
SwissADME plot of drug-likeness of cleomin. The pink area represents the optimal range for each property. Lipophilicity (LIPO): XLOGP3 between −0.7 and +5.0. SIZE: molecular weight between 150 and 500 g/mol. Polarity (POLAR): TPSA between 20 and 130 Å2. Insolubility (INSO): −6 < log S (ESOL) < 0. Insaturation (INSATU): fraction of carbons in the sp3 hybridization not less than 0.25. Flexibility (FLEX): no more than 9 rotatable bonds.

The SWISSADME analysis for cleomin indicates that the majority of its physicochemical properties fall within the desirable range, as denoted by the pink-shaded region for each parameter (Figure 9). Notably, cleomin exhibits a remarkable feature in terms of lipophilicity. This characteristic was assessed using various algorithms, including iLOGP, XLOGP3, WLOGP, MLOGP, and SILICOS-IT [12,13,14], yielding an average Log P(o/w) value of 1.41. In terms of pharmacokinetics, predictions do not exhibit inhibitory effects of cleomin on cytochrome P450 isoforms (1A2, 2C19, 2C9, 2D6, and 3A4). Concerning drug-likeness, cleomin adheres to Lipinski’s rules; however, its molecular weight (MW) of 145.22 g/mol falls just beyond the threshold stipulated by classical models such as Ghose and Muegge, which set a limitation of MW < 160 g/mol.

## 3. Discussion

The present study demonstrated, for the first time, that the intraperitoneal administration of cleomin at doses that did not induce motor impairment produced consistent antinociceptive effects. The data analysis from nociceptive tests suggests that the antinociceptive properties of cleomin may be associated with a blockade of neural pain transmission, such as that described in GABAergic and cholinergic pathways. Docking data corroborate this hypothesis. These results identify a new pharmacological property of cleomin, a 1,3-oxazolidine-2-thione compound isolated from *N. longifolium*. Therefore, thre is support for an ethnomedicinal use from this species for painful conditions.

The ethnomedicinal application of *Neocalyptrocalyx longifolium* aims to treat painful conditions such as abdominal pain. The occurrence of sedative effects in this plant species has also been reported [10]. However, it has not been established whether part of this effect can be attributed to cleomin. Thus, the first test performed was the rota-rod, in order to define the cleomin doses that do not induce motor impairment. This evaluation is essential to avoid misinterpretation of the results since the reduction in the behavioral nociceptive response induced by drugs could be associated with motor deficits. The induction of motor impairment effects occurred from a dose of 50 mg/kg, and based on this result, the cleomin doses tested in the nociceptive tests were equal to or below 25 mg/kg.

The antinociceptive properties of cleomin were initially investigated in the formalin test in mice, a screening tool for the assessment of analgesic or anti-inflammatory properties of new substances. The phases of the formalin test may indicate different types of pain and, therefore, this test is useful not only for identifying analgesic compounds but also for highlighting the possible mechanism of analgesia [15]. The early phase is a result of direct stimulation of the nociceptors, while the late phase is caused by local inflammation with a release of inflammatory and hyperalgesic mediators [15,16]. A decrease in licking time in both formalin phases is characteristic of drugs, such as opioid analgesics, that block the neural transmission of pain. In contrast, anti-inflammatory drugs suppress only the second phase of the formalin test [17]. The intraperitoneal administration of cleomin produced a significant antinociceptive effect in both the early and late phases of the test. This effect profile suggests that cleomin-induced antinociception involves the modulation of pain transmission rather than anti-inflammatory action.

To investigate this hypothesis, cleomin was next evaluated in the cold plate and tail flick tests, which mainly identify central analgesics. In the cold plate test, the intraperitoneal administration of cleomin reduced the nociceptive behavior at 1 and 2 h after treatment. Reinforcing this idea, cleomin also exhibited antinociceptive activity in the tail flick test. The thermal nociceptive stimulus in the tail flick test involves a spinal reflex, in addition to other higher neural structures [17,18]. In the cold plate test, cold hypersensitivity is an important feature of pain. The animal’s response to the nociceptive events involved in the test mimics the central processing of cold stimuli, which is considered a supraspinal-integrated response [19,20]. Thus, both become useful strategies to explore and investigate the site of action of antinociceptive agents with central actions, given their efficiency and specificity. These results indicate that the antinociceptive effect of cleomin may have a centrally mediated component. Reinforcing this idea, data from the estimated permeation model applied in SWISSADME, which considers the structural features of molecules to predict their permeability through biological membranes, suggested that cleomin can efficiently cross the blood–brain barrier. On the other hand, proving the hypothesis that cleomin induces analgesia through central action still requires further studies.

Next, the involvement of pain-modulating systems in the antinociceptive action of cleomin was evaluated. To achieve this goal, the effects of the pharmacological blockade of opioid, serotonergic, cholinergic, α-adrenergic, and GABAergic receptors on cleomin-induced antinociception were evaluated. Interestingly, only the antagonists of GABA_B_ (phaclofen) and muscarinic (atropine) receptors reduced the antinociceptive effects of cleomin in both nociceptive tests. It is important to highlight that although phaclofen and atropine promoted a reduction of the antinociceptive effects of cleomin in both nociceptive tests, this was more evident in the cold plate test. As previously described, the tail flick and cold plate tests measure responses processed at different levels of the central nervous system that may involve different neurotransmission systems, which may explain the variation in their sensitivity to the effects of cleomin.

It is well established that the muscarinic and GABA_B_ receptors are involved in the processing of pain signals and, thus, they are considered a valuable target for the generation of new analgesics. Cholinergic activation provides pain control by releasing analgesic modulators and changing the permeability of various ion channels through supraspinal mechanisms. GABA, in turn, modulates nociceptive transmission at the dorsal horn of the spinal cord level through the activation of GABA_A_ and GABA_B_ receptors that are located on primary afferent terminals as well as on dorsal horn neurons [21]. Corroborating the data from the functional antagonism assays, docking studies were performed to show the possible interactions of cleomin with muscarinic (M2) and GABA_B_ receptors.

A docking study is the application of computer-based models to predict the best fit orientation of a molecule into a determined macromolecular target [22]. The interactions of small molecules with their physiological receptors play a pivotal role in pharmacological activity. Based on that, docking analysis allows us to characterize the behavior of selected compounds in the binding site of target proteins as well as to elucidate fundamental biochemical processes [23], which aid in the discovery and comprehension of mechanisms of actions.

Sulfur-containing substances are commonly found in marine organisms [24]. In terrestrial plants, their occurrence is limited to few species, making 1,3-oxazolidine-2-thiones rare natural products. Analyzing the mode of interaction of cleomin through molecular docking, it was possible to verify that heterocyclic rings containing oxygen atoms are important structural features related to the activity of cleomin on the muscarinic receptor, as this compound showed similar interactions with the 4,5-dihydroisoxazole ring of iperoxo. On the other hand, in the GABA_B_ active site, the interactions seem to rely on different elements. Among them, the presence of a sulfur atom and alkyl moieties allowed for hydrogen bonding and other interactions with critical residues, which may contribute to the modulation of the antinociceptive activity by the 1,3-oxazolidine-2-thione core.

Both M2 and GABA_B_ are metabotropic receptors coupled to Gi/o proteins that mediate adenylyl cyclase inhibition [25,26,27]. This leads to the opening of rectifier potassium channels, resulting in hyperpolarization of the neuronal membrane, thereby raising the threshold for action potential generation [27,28]. Furthermore, GABA_B_ receptors are also located on presynaptic neurons. This activation results in the blockage of voltage-gated Ca^2+^ channels, thereby reducing neurotransmitter release [29]. Through these mechanisms, both receptors control the excitability and activity of nociceptive neurons, thus modulating events involved in pain transmission [25,26].

A promising pharmacological property of cleomin is the ability to induce antinociception in a lower dose range than that required to induce sedation. The sedative effects of analgesics, such as opioids and gabapentinoids, are already identified at preclinical analgesic doses [30,31]. This distinct pharmacological profile can be justified by the difference in the mechanism of action of cleomin in relation to these analgesics, as suggested by the functional antagonism assays and molecular docking studies. The fact that cleomin induces analgesia without identifiable sedation in the preclinical stage highlights its potential as a candidate for the drug discovery process.

In line with this idea, in silico pharmacokinetics prediction showed a good druggability profile of cleomin. SWISSADME analysis for cleomin indicated excellent lipophilicity properties. The significance of this observation extends to cleomin’s pharmacokinetics and bioavailability, as lipophilicity is closely associated with its ability to passively traverse the gastrointestinal tract and the blood–brain barrier. The presence of the blood–brain barrier exerts influence over the pharmacotherapy of drugs that necessitate access to the central nervous system. Given this context, the use of computational prediction models proves to be a time- and effort-saving approach, obviating the need for extensive experimental evaluations [32]. According to the estimated permeation model applied in SWISSADME [33], cleomin exhibits a high likelihood of being absorbed efficiently by the gastrointestinal tract and penetrating the blood–brain barrier. In addition, in silico pharmacokinetics predictions do not exhibit inhibitory effects of cleomin on cytochrome P450 isoforms, thus reducing the potential for drug–drug interactions.

## 4. Materials and Methods

### 4.1. Animals

Experiments were performed on male Swiss mice (20–23 g) obtained from the animal facilities of the Gonçalo Moniz Institute/FIOCRUZ (Salvador, BA, Brazil). Mice were housed under a 12:12 h dark–light cycle. Animals had access to water and food ad libitum and were housed in temperature-controlled rooms (22–25 °C). All behavioral tests were performed between 8:00 a.m. and 5:00 p.m. Each mouse was only tested once. Animal care and handling procedures were conducted in accordance with the National Institutes of Health guide for the care and use of laboratory animals (NIH, 8023). This research was approved by the Institutional Animal Care and Use Committee FIOCRUZ (IGM 025/2017). Every effort was made to minimize the number of animals used and to avoid any unnecessary discomfort.

### 4.2. Extraction and Isolation

Cleomin was isolated from the root bark of *Neocalyptrocalyx longifolium* (Capparaceae) collected in June 2018 in Monteiro, Paraíba, Brazil (7.8888° S, 37.1171° W). The plant was identified by Dr. José Iranildo Miranda, from the Herbarium Manuel de Arruda Câmara of the State University of Paraíba, Brazil, where a voucher specimen (HACAM 971) was deposited. The use of cleomin for research purposes was registered in the National Genetic Heritage Management System (SisGen, permit number A885D6F). Cleomin was purified as previously described [9]. The percent purity of cleomin used in the pharmacological experiments was greater than 98%, as determined via high-performance liquid chromatography.

### 4.3. Motor Function Assay

To evaluate possible nonspecific muscle-relaxant or sedative effects of cleomin, mice were subjected to the rota-rod test, as previously described [34]. The rota-rod apparatus (Insight, Ribeirão Preto, Brazil) consists of a bar with a diameter of 3 cm, subdivided into five compartments. The bar rotates at a constant speed of 6 revolutions per minute. Animals were selected 24 h prior to the experiment by excluding those mice that did not remain on the bar for two consecutive periods of 120 s. On the test day, mice were intraperitoneally (i.p.) treated with cleomin (25–100 mg/kg), vehicle (5% DMSO in 0.9% saline; control group), or intraperitoneally (i.p.) treated with diazepam (10 mg/kg, reference drug). After 40 min, mice were placed on the rotating rod, and the resistance to falling was measured for up to 120 s. The results were expressed as the average time (s) the animals remained on the rota-rod in each group.

### 4.4. Formalin Test

Mice were placed in an open Plexiglas observation chamber for 30 min to acclimate to their surroundings and then removed for formalin administration. Mice were gently restrained while 20 μL of 2.5% formalin (1:100 dilution of stock formalin solution, 37% formaldehyde in 0.9% saline) was administered subcutaneously to the plantar surface of the hind paw using a 30-gauge needle. Following injection, mice were returned to the observation chamber for a 30 min observation period. A mirror was placed behind the chamber to enable unhindered observation of the formalin-injected paw. The formalin injection produces a biphasic behavioral reaction [35]. The early phase is related to the direct stimulation of nociceptors and is sensitive to local anesthetics and opioids, whereas the late phase involves both inflammatory mediators and central sensitization and responds to opiates and anti-inflammatory drugs. Therefore, mice were observed from 0 to 10 min (early phase) and from 10 to 30 min (late phase), and a nociception score was determined for each period by counting the time that the animal spent licking the injected paw during the observation time [36]. Mice were intraperitoneally treated with cleomin (6.25–25 mg/kg), vehicle (5% DMSO in 0.9% saline; control group), indomethacin (10 mg/kg; reference drug), or morphine (5 mg/kg; reference drug) 40 min before formalin administration.

### 4.5. Cold Plate Test

The cold thermal nociceptive threshold was evaluated in a cold plate device (Teca^®^, Chicago, IL, USA) at a temperature of −2.5 ± 0.2 °C. Mice were kept for five minutes on the cold plate, and the nociceptive response was quantified by counting the nociceptive behaviors, namely hind paw lifts, hind paw lickings, flinches, and jumps [37]. The animals were acclimated to the cold plate the day before the test, remaining for 2 min at the same temperature used in the test. The results were expressed as nociception index, which represents the total amount of nociceptive responses in 5 min. The number of nociceptive events was measured before (baseline) and after treatments. Mice were intraperitoneally treated with cleomin (6.25–25 mg/kg), vehicle (5% DMSO in 0.9% saline; control group), or morphine (5 mg/kg, reference drug).

### 4.6. Tail Flick Test

The tail flick test in mice was conducted as described [36]. Before the experiment, each animal was habituated to the restraint cylinder for 20 min/day for 5 consecutive days. On the day of the experiment, mice were placed in the restraint cylinder, and the tail tip (2 cm) was submerged in a water bath at 48 ± 0.5 °C. The latency of the tail withdrawal reflex was measured. Each submersion was terminated after 10 s to minimize potential skin damage. Tail flick latency was measured before (baseline) and after treatments. Mice were intraperitoneally treated with cleomin (6.25–25 mg/kg), vehicle (5% DMSO in 0.9% saline; control group), or morphine (5 mg/kg, reference drug).

### 4.7. Functional Antagonism Assays

Further experiments were carried out to elucidate the possible mechanisms by which cleomin exerts its antinociceptive action. Antagonism assays were conducted employing tail flick and cold plate tests, using the maximum effective dose of cleomin (25 mg/kg, intraperitoneal route). Mice were intraperitoneally pretreated with different antagonists or saline and then received cleomin. The nociceptive response was recorded from 0.5 or 1 h after cleomin administration in the tail flick and cold plate tests, respectively. In the control group mice were treated with vehicle (5% DMSO in 0.9% saline) instead of cleomin. The following antagonists were tested: non-selective opioid antagonist, naloxone (5 mg/kg, 15 min before cleomin) [38]; a selective α2-adrenergic antagonist, yohimbine (2 mg/kg, 30 min before cleomin) [39]; gamma aminobutyric acid-A GABA_A_ receptor antagonist, bicuculline (1 mg/kg, 15 min before cleomin) [38]; gamma aminobutyric acid-B GABA_B_ receptor antagonist, phaclofen (2 mg/kg, 15 min before cleomin) [40]; non-selective serotonergic antagonist, methysergide (5 mg/kg, 30 min before cleomin) [39]; and non-selective cholinergic antagonist, atropine (10 mg/kg, 15 min before cleomin) [38]. The dose, route of administration, and pretreatment time for each antagonist studied were selected based on data from the literature, ensuring their optimal effect, and are summarized in Table 1. The tested drugs were purchased from Sigma-Aldrich (St Louis, MO, USA), except for naloxone, diazepam, and morphine, which were purchased from Cristália (Itapira, São Paulo, Brazil).

### 4.8. Docking Studies

Molegro Virtual Docker version 6.0 software was used to generate 20 poses for each molecule in the active site of the protein. The pose with the lowest interaction energy was selected and imported to Discovery Studio 2021 to visualize the results. The Moldock scoring algorithm was used, along with the Moldock search algorithm [41,42]. The targets (PDB 4MQS and 4MS3) [43,44] and cleomin structure were prepared using the parameter settings in the software package (ligand evaluation: Internal ES, Internal H-Bond, Sp2-Sp2 Torsions, all checked; number of runs: 20 runs; algorithm: MolDock SE; maximum interactions: 2.000; Max. population size: 70; Max. steps: 300; neighbor distance factor: 1.00; Max. number of poses returned: 15).

Docking results were validated by redocking the crystallographic ligand with both investigated proteins. Root Mean Square Deviations (RMSDs) lower than 2 Å indicate the optimal degree of screening reliability. To acquire more reliable result comparison, the energy values obtained from docked molecules were normalized by molecular mass [45], as demonstrated in the following equation:

(1) $$E_{N}=E_{KJ/mol\;}÷MW$$

where *E_N_* is the normalized value; *E_KJ/mol_* is the docking energy of the compounds; and *MW* is the molecular mass of each compound (cleomin and the crystallographic ligand of each target).

### 4.9. In Silico Pharmacokinetics Prediction of Cleomin

SwissADME was applied to assess the druggability of cleomin [46]. SwissADME is a web application that offers reliable predictive models for the pharmacokinetics and drug-likeness of synthetic and natural compounds with pharmacological interest based on their structural features [47,48,49]. As input data, the SMILES (Simplified Molecular Input Line Entry System) of the compound were used and then submitted to analysis.

### 4.10. Statistical Analysis

Data were presented as means ± standard deviation (SD) of measurements made on six animals in each group. Comparisons among three or more groups were made using one-way ANOVA followed by Tukey’s post hoc test or, for repeated measures, two-way ANOVA followed by Bonferroni’s post hoc test. All data were analyzed using the program Prism 9. Differences were considered statistically significant for *p* values < 0.05.

## 5. Conclusions

Taken together, the results of the present study show that cleomin promotes antinociception, mediated, at least in part, by GABA_B_ and muscarinic receptors. This is the first demonstration of the pharmacological properties of cleomin that supports the ethnomedicinal use of *N. longifolium* for the treatment of pain. This work demonstrates the strong potential of this 1,3-oxazolidine-2-thione and highlights it as a promising candidate for treating painful conditions and deserves particular attention in further research and development.

## Figures and Tables

**Table 1:** Substances used on functional antagonism assays.

Antagonist	Dose/via	Pre-Treatment Time	References
Naloxone	5 mg/kg/ip.	15 min	[38]
Yohimbine	2 mg/kg/ip.	30 min	[39]
Methysergide maleate	5 mg/kg/ip.	30 min	[39]
Atropine	10 mg/kg/ip.	15 min	[38]
Bicuculline	1 mg/kg/ip.	15 min	[38]
Phaclofen	2 mg/kg/ip.	15 min	[40]

## Data Availability

The data presented in this study are available on request from the corresponding author.
